# Association between drug intake and incidence of malignancies in patients with Juvenile Idiopathic Arthritis: a nested case–control study

**DOI:** 10.1186/s12969-016-0066-8

**Published:** 2016-02-03

**Authors:** Swaantje Barth, Jenny Schlichtiger, Betty Bisdorff, Boris Hügle, Hartmut Michels, Katja Radon, Johannes-Peter Haas

**Affiliations:** Institute and Outpatient Clinic for Occupational, Social and Environmental Medicine, University Hospital of Munich (Ludwig-Maximilans-University), Ziemssenstr.1, 80337 Munich, Germany; German Center for Pediatric and Adolescent Rheumatology, Gehfeldstraße 24, 82467 Garmisch-Partenkirchen, Germany; Occupational and Environmental Epidemiology & NetTeaching Unit, Institute and Outpatient Clinic for Occupational, Social and Environmental Medicine University Hospital of Munich (Ludwig-Maximilians-University), Ziemssenstr.1, 80336 Munich, Germany

**Keywords:** Juvenile idiopathic arthritis (JIA), Malignancy, Cancer, Incidence, Drug intake, Biologics, Tumour necrosis factor α

## Abstract

**Background:**

Several medications for treatment of Juvenile Idiopathic Arthritis (JIA) are considered to be carcinogenic. Therefore, the aim was to assess whether there is an association between therapeutic interventions and malignancies in JIA patients.

**Findings:**

A nested case–control study was carried out within a retrospective cohort study of 3698 JIA patients diagnosed between 1952 and 2010. All 48 JIA patients with a diagnosis of a malignant tumour and up to four matched controls for each received a questionnaire about their use of medication. Subsequently treatment was compared between cases and controls and analyses performed for 37 cases and 125 controls (response 88.5 %). Treatment with DMARD (84 %) was most frequently used, followed by glucocorticoids (66 %) and immunosuppressives (65 %). Twenty percent reported to have ever been taking biologics. Medication use did not differ significantly between cases and controls.

**Conclusions:**

Our results did not show an association between medications used and malignancies in JIA patients.

**Electronic supplementary material:**

The online version of this article (doi:10.1186/s12969-016-0066-8) contains supplementary material, which is available to authorized users.

## Findings

### Introduction

Patients with Juvenile Idiopathic Arthritis (JIA) are considered to be at higher risk of malignancies than the general population [1]. Potential explanations for this increased risk might be the inflammatory process underlying the disease [2], frequent use of diagnostic procedures involving exposure to ionizing radiation [3] or therapeutic interventions [1].

Over the last two decades, new therapeutic options seem to have considerably improved treatment and outcome [4–7]. Substantial therapeutic improvement was achieved through the introduction of biologic agents [4] such as Tumour-necrosis-factor-alpha (TNFα)-blockers for example [4, 8]. However, concerns about these medications were first risen in 2008 by the Food and Drug Administration (FDA) resulting in a warning about a possible association between the use of TNFα-blockers and the development of malignancies in children and young adults [9]. However, since subsequent studies could not confirm such an association for patients with JIA treated with TNFα-blockers, it has been suggested that the association between TNFα-blockers and malignancy might be confounded by the concomitant intake of immunosuppressive agents [10] or by the inflammatory process itself [2, 11].

Most recently published reviews on the subject matter concluded that an association between use of biologics and malignancies is unlikely, but that more long-term studies are needed to be able to draw definite conclusions [1, 4, 12]. Therefore, this study carried out a retrospective cohort study on cancer incidence in 3692 JIA patients (Barth S, Schlichtiger J, Hartmann B, Bisdorff B, Michels H, Radon K et al. Incidence of malignancies in patients with Juvenile Idiopathic Arthritis: a retrospective single-centre cohort study,submitted.) Nested within this retrospective cohort study, a case–control study to investigate whether type of treatment differed between JIA patients with and without cancer was also performed.

## Methods

### Study design and study population

A nested case–control study was carried out within of a retrospective single-centred hospital-based cohort study of 3698 JIA patients. In 2012, all current and former JIA patients that had been admitted to the German Centre for Pediatric and Adolescent Rheumatology (GCPAR) in Garmisch-Partenkirchen (Germany) between 1952 and 2010 (*n* = 10,580) were sent a self-administered standardized questionnaire. Overall, 6127 completed the questionnaire and gave written informed consent. As described elsewhere (Barth S, Schlichtiger J, Hartmann B, Bisdorff B, Michels H, Radon K et al. Incidence of malignancies in patients with Juvenile Idiopathic Arthritis: a retrospective single-centre cohort study,submitted), by means of reviewing the actual medical records at the GCPAR, JIA patients were identified and those suffering from other (rheumatic) diseases excluded (Barth S, Schlichtiger J, Hartmann B, Bisdorff B, Michels H, Radon K et al. Incidence of malignancies in patients with Juvenile Idiopathic Arthritis: a retrospective single-centre cohort study,submitted). Of the remaining participants, all patients reporting a malignant tumour were defined as cases (International Classification of Diseases, Ninth Revision (ICD9) 140–208). Subsequently, a total of 48 cases were individually matched by sex, age (+/− 2 years) and date of first admission to the hospital (+/− 2 years) with up to four controls each. These were selected out of the JIA cohort from the same study. In the spring of 2013, all cases and 135 controls received a second self-administered standardized questionnaire including informed consent. The study was approved by the medical ethics committee of the University Hospital of Munich (LMU) in September 2011.

### Questionnaire

A 19-item self-administered questionnaire (copy available from the authors upon request) was used to assess:

- Comorbidities (diabetes, HIV, mononucleosis, Crohn's disease, ulcerative colitis, multiple sclerosis, Hashimoto's thyroiditis, and trisomy 21),
- Drug intake (abatacept, adalimumab, anakinra, azathioprine, cyclophosphamide, chlorambucil, chloroquine, cyclosporine a, D-penicillamine, etanercept, hydroxychloroquine, infliximab, leflunomide, methotrexate, mycophenolatmofetil, natriumaurothiomalate, oral cortisone, rituximab, sulfasalazine, tocilizumab),
- If a specific type of drug was taken, duration of intake was requested (<6 month, 6 month-2 years, >2 years),
- Potential other risk factors of cancer (parental cancer history, computed tomography (CT), x-ray, scintigraphy, nuclear radiation therapy).

In addition, sociodemographic data (age, sex, education of participants and their parents) and disease-related information (disease duration and age at first symptoms of JIA) were taken from the cohort questionnaire that had previously been filled out (Barth S, Schlichtiger J, Hartmann B, Bisdorff B, Michels H, Radon K et al. Incidence of malignancies in patients with Juvenile Idiopathic Arthritis: a retrospective single-centre cohort study,submitted).

Data were double entered into SurveyMonkey (SurveyMonkey Inc., USA) and the entries checked with Synkronizer 10.0 (©2000–2014, XL Consulting GmbH, Switzerland).

### Variable definitions

The assessed drugs were categorized into following active ingredient groups:

- disease-modifying antirheumatic drugs (DMARDs) *(chloroquine, hydroxychloroquine, sulfasalazine, D-penicillamine, natriumaurothiomalate, methotrexate, cyclosporine a, leflunomide, mycophenolatmofetil, azathioprine)*
- cytostatics *(cyclophosphamide, chlorambucil)*
- glucocorticoids *(oral cortisone)*
- immunosuppressives *(methotrexate, cyclosporine a, leflunomide, mycophenolatmofetil, azathioprine, cyclophosphamide, chlorambucil)*
- biologic agents *(abatacept, anakinra, etanercept, infliximab, rituximab, tocilizumab, adalimumab)*

### Statistical analyses

Absolute and relative frequencies were used to describe categorical data. For continuous data, measures of central tendency (median values) and measures of dispersion (1^st^–3^rd^ quartile and range) were calculated. All analyses were stratified for cases and controls. Differences between cases and controls were identified using conditional (fixed-effect) logistic regression analyses; Odds Ratios (OR) and 95 %-Confidence Intervals (95 %-CI) were calculated. Due to the large numbers of missing values for drug intake, sensitivity analyses were performed. Multivariate analyses were repeated first including a missing category for the drug variables, followed by the coding of the missing values as “drug was not taken”. These results were compared then to the main analyses. Furthermore, drug intake was validated by sending a second questionnaire with the same questions on drug intake out again about a year later. By calculating Cramer`s V values we tested reliability of answers. All statistical analyses were performed using Stata software version 12.1 (StataCorp LP, USA).

## Results

The study population consisted of 48 cases and 135 controls with 37 cases and 125 controls responding (response 88.5 %). Median age of participants was 43 years (1^st^–3^rd^ quartile: 33–50 years; range: 12–67 years), 82 % were female and most participants had a medium (41 %) or high (38 %) level of education (Table 1). The most common type of cancer was melanoma (*n* = 9), followed by breast cancer (*n* = 7) and cervical cancer (*n* = 5). All other types of cancer occurred in three or fewer patients. Median duration from onset of JIA to diagnosis of the malignant tumour was 26 years (1st-3rd quartile: 21–34.5; range: 9–47 years).

**Table 1:** Socio-demographic characteristics and diseases-specific data of the study population

	n _missing_	Case n (%)	Control n (%)	OR (95 %-CI)^a^
Total		37 (22.8)	125 (77.2)	
Sex of the participant				
Male	0	7 (18.9)	23 (18.4)	n. a.
Female		30 (81.1)	102 (81.6)	
Age (years)				
0–17	0	2 (5.4)	8 (6.4)	n. a.
18–24		0 (0.0)	0 (0.0)	
25–34		7 (18.9)	28 (22.4)	
35–44		12 (32.4)	39 (31.2)	
45–54		10 (27.0)	36 (28.8)	
55–76		6 (16.2)	14 (11.2)	
Education of participant^b^				
Low	3	8 (21.6)	25 (20.5)	1.00 (Ref.)
Medium		14 (37.8)	51 (41.8)	0.90 (0.33;2.46)
High		15 (40.5)	46 (37.7)	1.03 (0.35;3.06)
Parental education				
Low	35	12 (44.4)	39 (39.0)	1.00 (Ref.)
Medium		9 (33.3)	35 (35.0)	0.85 (0.28;2.53)
High		6 (22.2)	26 (26.0)	1.18 (0.33;4.16)
Disease duration since first symptoms (years)	65			
Median (1^st^–3^rd^ quartile) (range)		41 (35;44) (3;57)	39 (34.5;45) (4;62)	0.97 (0.83;1.13)
Age at first symptoms (years)	65			
Median (1^st^–3^rd^ quartile) (range)		8.5 (5;11) (1;13)	8 (4;11) (0;15)	1.03 (0.90;1.19)

*OR* odds ratio, *CI* confidence interval, *n. a* not available, *DMARD* disease-modifying anti-rheumatic drugs, *CT* computed tomography

^a^OR of conditional (fixed-effects) logistic regression analysis with cancer (yes/no) as outcome. For each independent variable a separate model was created

^b^Level of education was summarized into high (higher school certificate, university, or college degree), medium (secondary school leaving certificate, or comparable degree) and low (lower secondary education level or no degree). For children, pupils and for those who did not indicate their level of education the highest parental level of education was used as a proxy

On average, cases and controls had exposure to three different drugs during the course of their disease (range: 0–17). Intake of any antirheumatic medication during the last 12 months was reported by 49 % (data not shown). Treatments most frequently included: oral cortisone (66 %), methotrexate (51 %), natriumaurothiomalate (47 %) and chloroquine (42 %). Steroids were taken by 39 % for more than two years thus being the medication with the longest duration of intake. Duration of intake and type of drugs did not differ between cases and controls (see Additional file 1).

Considering the categorized active ingredient groups, treatment most commonly included DMARD (84 %). More than half of all patients reported intake of glucocorticoids (66 %) and immunosuppressives (65 %). Biologics were ingested by 20 % of all respondents and cytostatics by 6 %. The intake of the investigated drugs was not statistically significant associated with being a case (Table 2) – not even in sensitivity analyses when the large number of missing values was considered (data not shown).

**Table 2:** Participants drug intake

	n _missing_ (%)	Case n (%)	Control n (%)	OR (95 %-CI)^a^
Total		37 (22.8)	125 (77.2)	
DMARD				
No	10 (6.17)	5 (14.3)	20 (17.1)	1.00 (Ref.)
Yes		30 (85.7)	97 (82.9)	1.25 (0.42;3.76)
Immunosuppressives				
No	21 (12.96)	11 (35.5)	39 (35.5)	1.00 (Ref.)
Yes		20 (64.5)	71 (64.6)	0.97 (0.42;2.27)
Cytostatics				
No	42 (25.93)	27 (93.1)	86 (94.5)	1.00 (Ref.)
Yes		2 (6.9)	5 (5.5)	1.29 (0.23;7.18)
Glucocorticoids				
No	26 (16.05)	7 (21.2)	39 (37.9)	1.00 (Ref.)
Yes		26 (78.8)	64 (62.1)	2.31 (0.88;6.10)
Biologics				
No	38 (23.46)	25 (86.2)	74 (77.9)	1.00 (Ref.)
Yes		4 (13.8)	21 (22.1)	0.75 (0.24;2.38)

*OR* odds ratio, *CI* confidence interval, *DMARD* disease-modifying anti-rheumatic drugs

^a^OR of conditional (fixed-effects) logistic regression analysis with cancer (yes/no) as outcome. For each independent variable a separate model was created

With regard to other potential factors, an association between exposure to scintigraphy and nuclear radiation therapy could be shown. However, in this context it has to be considered that both are used for the diagnosis of cancer. Therefore, with our data it is not possible to classify these as risk factors for cancer since application took place or simultaneously or after diagnosis of cancer. The remaining factors showed a tendency leading to (family history of cancer, x-ray) or having no impact on cancer (Table 3).

**Table 3:** Risk factors for cancer

	n _missing_	Case n (%)	Control n (%)	OR (95 %-CI)^a^
Total		37 (22.8)	125 (77.2)	
Parental cancer history				
No	3	18 (51.4)	78 (62.9)	1.00 (Ref.)
Yes		17 (48.6)	46 (37.1)	1.94 (0.92;4.13)
Ever Smoked				
No	1	16 (44.4)	66 (52.8)	1.00 (Ref.)
Yes		20 (55.6)	59 (47.2)	1.49 (0.66;3.41)
Currently smoking				
No	0	29 (78.4)	94 (75.2)	1.00 (Ref.)
Yes		8 (21.6)	31 (24.8)	0.84 (0.32;2.15)
CT				
No	0	9 (24.3)	60 (48.0)	1.00 (Ref.)
Yes		28 (75.7)	65 (52.0)	0.95 (0.51;1.76)
Scintigraphy				
No	0	17 (45.9)	91 (72.8)	1.00 (Ref.)
Yes		20 (54.1)	34 (27.2)	2.01 (1.15;3.50)
X-ray (dentist)				
0–5	20	16 (47.1)	46 (42.6)	1.0 (Ref.)
6–10		10 (29.4)	29 (26.9)	0.96 (0.37;2.48)
11–20		5 (14.7)	19 (17.6)	0.81 (0.25;2.69)
>20		3 (8.8)	14 (13.0)	0.65 (0.16;2.71)
X-ray				
0–5	15	1 (2.8)	11 (9.9)	1.00 (Ref.)
6–10		2 (5.6)	14 (12.6)	1.85 (0.15;22.58)
11–20		10 (27.8)	29 (26.1)	4.27 (0.46;39.39)
>20		23 (63.9)	57 (51.4)	4.34 (0.52;36.01)
Nuclear radiation therapy				
No	1	26 (70.3)	114 (91.9)	1.00 (Ref.)
Yes		11 (29.7)	10 (8.1)	3.04 (1.25;7.36)

*OR* odds ratio, *CI* confidence interval, ^a^OR of conditional (fixed-effects) logistic regression analysis with cancer (yes/no) as outcome

Validation of drug intake revealed moderate agreement with most of Cramer´s V values between 0.52 and 0.74 except for two, which showed lower agreement (anakinra: 0.29 and mycophenolatmofetil: 0.30).

## Discussion

This study compared drug intake and exposure to different already established cancer risk factors between JIA patients with and without malignancies. No statistical significant difference regarding drug intake was found. Only two of the already previously established risk factors were confirmed (exposure to scintigraphy, nuclear radiation therapy) in this study.

These results are in line with several previous studies that either found no increased incidence of malignancies in JIA patients [13–15] or an increased risk for cancer independent of TNF-α-treatment [1, 16–20]. However, some studies suggested that new therapeutics might be responsible for an increased risk of cancer in JIA patients [13, 21]. On the contrary it has been suggested that the use of biologic agents, among others, lowers the background risk of JIA for malignancies in reducing the inflammatory process of the disease [12].

Although, drug intake did not differ between cases and controls we found differences with regards to use of scintigraphy and nuclear radiation therapy, which was more often used in the cases. However, these results were to be expected since these methods are used for cancer diagnosis [22, 23].

For some variables we had a high number of missing values especially for date of first symptoms and drug intake; date of first symptoms was often unknown and is generally hard to define.

The reliability of information based on response to the questions on drug intake is a major limiting factor of this study, as it was too difficult for some of the patients to remember such detailed questions retrospectively. We used several methods in order to control for possible bias due to missing values. No statistically significant differences were found between patients with and without a missing value in one of the drug variables. Inclusion of missing values into multivariate analyses revealed no effect on having cancer, number of missing values did not differ between cases and controls.

On one hand the long recruitment period can be considered a strength of the study,but simultaneously it might also have lead to a possible recall bias.

Especially, in the context of drug intake recall bias cannot be excluded as the results of drug validation showed only moderate agreement between first and second drug questionnaire. Therefore, the results should be interpreted with caution. Validation of drug intake with medical records was anticipated, but in the end was not possible as most patients are now being treated elsewhere. Therefore, obtaining all medical records of the patients was not feasible in the context of this study.

Lower the number of missing values was not successful not even by phoning participants up; most frequently they stated that they could not remember the name of the drugs. Multiple medications were not considered as a potential risk factor for cancer in this study but might well be relevant for future studies. Another obvious restriction of the study was the small number of cases. However, with regards to the total number of cases in the first part of the study 90 % of all cases were reached for the follow-up. Nevertheless, to draw valid conclusions for the differences between cases and controls regarding drug treatment and other environmental cancer risk factors larger case–control-studies are needed. Therefore in this study it has to be considered that missing differences between cases and controls were due to missing power.

In conclusion, this study on cancer cases from a large single-centered population over a time period of more than half a century, could not suggest an increased risk of malignancies in JIA patients associated with JIA therapy.

## Additional file

Additional file 1:
**Intake and duration of rheumatic drugs.** (DOCX 28 kb)

## Abbreviations

95 %-CI: 95 %-Confidence Intervals
DMARDs: disease-modifying antirheumatic drugs
FDA: Food and Drug Administration
GCPAR: German Centre for Pediatric and Adolescent Rheumatology
ICD9: International Classification of Diseases, Ninth Revision
JIA: Juvenile Idiopathic Arthritis (JIA)
OR: Odds Ratios
TNFα-blockers: tumour-necrosis-factor-alpha blockers
