# Supplementary material for: Association between drug intake and incidence of malignancies in patients with Juvenile Idiopathic Arthritis: a nested case–control study
Source: Pediatr Rheumatol Online J. 2016 Feb 3;14:6. doi: 10.1186/s12969-016-0066-8 (PMC4739096; doi:10.1186/s12969-016-0066-8)
Supplement: Additional file 1: — Intake and duration of rheumatic drugs. (DOCX 28 kb) [file 12969_2016_66_MOESM1_ESM.docx]

Table A1: Intake and duration of rheumatic drugs

|  | Drug intake | | | | | Duration of drug intake | | | | | |
| --- | --- | --- | --- | --- | --- | --- | --- | --- | --- | --- | --- |
| Drug | n _missing_ | Case  n (%) | Control  n (%) | Total  n (%) | OR (95%-CI)^1^ | Drug | n _missing_ | Case  n (%) | Control  n (%) | Total  n (%) | OR (95%-CI)^1^ |
| Oral cortisone  Yes | 26 | 26 (78.8) | 64 (62.1) | **90 (66.2)** | 2.31 (0.88;6.10) | Oral cortisone  Never  < 6 month  6 month – 2 years  > 2 years | 27 | 7 (21.9)  5 (15.6)  5 (15.6)  15 (46.9) | 39 (37.9)  15 (14.6)  12 (11.7)  37 (35.9) | 46 (34.1)  20 (14.8)  17 (12.6)  **52 (38.5)** | 1.27 (0.91; 1.76) |
| Methotrexate  Yes | 27 | 16 (51.6) | 53 (51.0) | **69 (51.1)** | 1.09 (0.50;2.38) | Methotrexate  Never  < 6 month  6 month – 2 years  > 2 years | 28 | 15 (50.0)  2 (6.7)  3 (10.0)  10 (33.3) | 51 (49.0)  3 (2.9)  17 (16.4)  33 (31.7) | 66 (49.3)  5 (3.7)  20 (14.9)  **43 (32.1)** | 1.01 (0.76; 1.35) |
| Natriumaurothiomalate  Yes | 27 | 15 (46.9) | 49 (47.6) | **64 (47.4)** | 1.06 (0.42;2.62) | Natriumaurothiomalate  Never  < 6 month  6 month – 2 years  > 2 years | 30 | 17 (54.8)  4 (12.9)  5 (16.1)  5 (16.1) | 54 (53.5)  11 (10.9)  15 (14.9)  21 (20.8) | 71 (53.8)  15 (11.4)  20 (15.2)  **26 (19.7)** | 0.93 (0.63; 1.37) |
| Chloroquine  Yes | 29 | 16 (50.0) | 40 (39.6) | **56 (42.1)** | 1.74 (0.76; 3.98) | Chloroquine  Never  < 6 month  6 month – 2 years  > 2 years | 30 | 16 (51.6)  2 (6.5)  8 (25.8)  5 (16.1) | 61 (60.4)  5 (5.0)  17 (16.8)  18 (17.8) | 77 (58.3)  7 (5.3)  25 (18.9)  **23 (17.4)** | 1.16 (0.82;1.63) |
| Azathioprine  Yes | 32 | 12 (40.0) | 39 (39.0) | **51 (39.2)** | 0.97 (0.38; 2.52) | Azathioprine  Never  < 6 month  6 month – 2 years  > 2 years | 35 | 18 (62.1)  2 (6.9)  3 (10.3)  6 (20.7) | 61 (62.2)  5 (5.1)  12 (12.2)  20 (20.4) | 79 (62.2)  7 (5.5)  15 (11.8)  **26 (20.5)** | 1.00 (0.69;1.46) |
| Sulfasalazine  Yes | 37 | 11 (37.9) | 36 (37.5) | **47 (37.6)** | 0.93 (0.38;2.28) | Sulfasalazine  Never  < 6 month  6 month – 2 years  > 2 years | 39 | 18 (64.3)  3 (10.7)  1 (3.6)  6 (21.4) | 60 (63.2)  5 (5.3)  12 (12.6)  18 (19.0) | 78 (63.4)  8 (6.5)  13 (10.6)  **24 (19.5)** | 0.97 (0.67; 1.41) |
| D-Penicillamine  Yes | 39 | 8 (27.6) | 21 (22.3) | **29 (23.6)** | 1.39 (0.48;3.97) | D-Penicillamine  Never  < 6 month  6 month – 2 years  > 2 years | 39 | 21 (72.4)  1 (3.5)  2 (6.9)  5 (17.2) | 73 (77.7)  5 (5.3)  8 (8.5)  8 (8.5) | 94 (76.4)  6 (4.9)  10 (8.1)  **13 (10.6)** | 1.19 (0.79; 1.79) |

n. a.: not available; ^1^ OR of conditional (fixed-effects) logistic regression analysis with cancer (yes/no) as outcome. For each independent variable a separate model was created.

cont. Table A1: Intake and duration of rheumatic drugs

|  | Drug intake | | | | | Duration of drug intake | | | | | |
| --- | --- | --- | --- | --- | --- | --- | --- | --- | --- | --- | --- |
| Drug | n _missing_ | Case  n (%) | Control  n (%) | Total  n (%) | OR (95%-CI)^1^ | Drug | n _missing_ | Case  n (%) | Control  n (%) | Total  n (%) | OR (95%-CI)^1^ |
| Leflunomide  Yes | 41 | 6 (20.7) | 21 (22.8) | **27 (22.3)** | 0.83 (0.28;2.50) | Leflunomide  Never  < 6 month  6 month – 2 years  > 2 years | 41 | 23 (79.3)  1 (3.5)  2 (6.9)  3 (10.3) | 71 (77.2)  4 (4.4)  7 (7.6)  10 (10.9) | 94 (77.7)  5 (4.1)  9 (7.4)  **13 (10.7)** | 0.96 (0.63;1.47) |
| Etanercept  Yes | 41 | 3 (10.3) | 16 (17.4) | **19 (15.7)** | 0.70 (0.18;2.63) | Etanercept  Never  < 6 month  6 month – 2 years  > 2 years | 41 | 26 (89.7)  0 (0.0)  0 (0.0)  3 (10.3) | 76 (82.6)  1 (1.1)  5 (5.4)  10 (10.9) | 102 (84.3)  1 (0.8)  5 (4.1)  **13 (10.7)** | 0.94 (0.58;1.51) |
| Hydroxychloroquine  Yes | 40 | 3 (10.3) | 14 (15.1) | **17 (13.9)** | 0.76 (0.17;3.34) | Hydroxychloroquine  Never  < 6 month  6 month – 2 years  > 2 years | 40 | 26 (89.7)  1 (3.5)  1 (3.5)  1 (3.5) | 79 (85.0)  4 (4.3)  9 (9.7)  1 (1.1) | 105 (86.1)  5 (4.1)  10 (8.2)  **2 (1.6)** | 0.92 (0.43; 1.98) |
| Adalimumab  Yes | 60 | 2 (10.0) | 6 (15.0) | **8 (13.3)** | n. a. | Adalimumab  Never  < 6 month  6 month – 2 years  > 2 years | 60 | 18 (90.0)  0 (0.0)  0 (0.0)  2 (10.0) | 34 (85.0)  3 (7.5)  1 (2.5)  2 (5.0) | 52 (86.7)  3 (5.0)  1 (1.7)  **4 (6.7)** | n. a. |
| Cyclosporine A  Yes | 44 | 1 (3.5) | 8 (9.0) | **9 (7.6)** | 0.47 (0.05;4.19) | Cyclosporine A  Never  < 6 month  6 month – 2 years  > 2 years | 44 | 28 (96.6)  0 (0.0)  0 (0.0)  1 (3.5) | 81 (91.0)  2 (2.3)  2 (2.3)  4 (4.5) | 109 (92.4)  2 (1.7)  2 (1.7)  **5 (4.2)** | 0.86 (0.38; 1.94) |
| Infliximab  Yes | 43 | 0 (0.0) | 4 (4.4) | **4 (3.4)** | n. a. | Infliximab  Never  < 6 month  6 month – 2 years  > 2 years | 44 | 29 (100)  0 (0.0)  0 (0.0)  0 (0.0) | 86 (96.6)  1 (1.1)  2 (2.3)  0 (0.0) | 115 (97.5)  1 (0.9)  2 (1.7)  **0 (0.0)** | n. a. |
| Chlorambucil  Yes | 42 | 0 (0.0) | 4 (4.40) | **4 (3.3)** | n. a. | Chlorambucil  Never  < 6 month  6 month – 2 years  > 2 years | 42 | 29 (100)  0 (0.0)  0 (0.0)  0 (0.0) | 87 (95.6)  2 (2.2)  2 (2.2)  0 (0.0) | 116 (96.7)  2 (1.7)  2 (1.7)  **0 (0.0)** | n. a. |

n. a.: not available; ^1^ OR of conditional (fixed-effects) logistic regression analysis with cancer (yes/no) as outcome. For each independent variable a separate model was created.

cont. Table A1: Intake and duration of rheumatic drugs

|  | Drug intake | | | | | Duration of drug intake | | | | | |
| --- | --- | --- | --- | --- | --- | --- | --- | --- | --- | --- | --- |
| Drug | n _missing_ | Case  n (%) | Control  n (%) | Total  n (%) | OR (95%-CI)^1^ | Drug | n _missing_ | Case  n (%) | Control  n (%) | Total  n (%) | OR (95%-CI)^1^ |
| Cyclophosphamide  Yes | 44 | 1 (3.6) | 2 (2.2) | **3 (2.5)** | 1.65 (0.15;18.24) | Cyclophosphamide  Never  < 6 month  6 month – 2 years  > 2 years | 44 | 27 (96.4)  1 (3.6)  0 (0.0)  0 (0.0) | 88 (97.8)  1 (1.1)  0 (0.0)  1 (1.1) | 115 (97.5)  2 (1.7)  0 (0.0)  **1 (0.9)** | 0.90 (0.20;3.97) |
| Rituximab  Yes | 44 | 1 (3.5) | 1 (1.1) | **2 (1.7)** | 2.45 (0.15;39.72) | Rituximab  Never  < 6 month  6 month – 2 years  > 2 years | 44 | 28 (96.6)  1 (3.5)  0 (0.0)  0 (0.0) | 88 (98.9)  0 (0.0)  0 (0.0)  1 (1.1) | 116 (98.3)  1 (0.9)  0 (0.0)  **1 (0.9)** | 0.96 (0.22;4.14) |
| Mycophenolatmofetil  Yes | 44 | 0 (0.0) | 0 (0.0) | **0 (0.0)** | n. a. | Mycophenolatmofetil  Never  < 6 month  6 month – 2 years  > 2 years | 44 | 29 (100)  0 (0.0)  0 (0.0)  0 (0.0) | 89 (100)  0 (0.0)  0 (0.0)  0 (0.0) | 118 (100)  0 (0.0)  0 (0.0)  **0 (0.0)** | n. a. |
| Abatacept  Yes | 44 | 0 (0.0) | 0 (0.0) | **0 (0.0)** | n. a. | Abatacept  Never  < 6 month  6 month – 2 years  > 2 years | 44 | 29 (100)  0 (0.0)  0 (0.0)  0 (0.0) | 89 (100)  0 (0.0)  0 (0.0)  0 (0.0) | 118 (100)  0 (0.0)  0 (0.0)  **0 (0.0)** | n. a. |
| Anakinra  Yes | 44 | 0 (0.0) | 0 (0.0) | **0 (0.0)** | n. a. | Anakinra  Never  < 6 month  6 month – 2 years  > 2 years | 44 | 29 (100)  0 (0.0)  0 (0.0)  0 (0.0) | 89 (100)  0 (0.0)  0 (0.0)  0 (0.0) | 118 (100)  0 (0.0)  0 (0.0)  **0 (0.0)** | n. a. |
| Tocilizumab  Yes | 44 | 0 (0.0) | 0 (0.0) | **0 (0.0)** | n. a. | Tocilizumab  Never  < 6 month  6 month – 2 years  > 2 years | 44 | 29 (100)  0 (0.0)  0 (0.0)  0 (0.0) | 89 (100)  0 (0.0)  0 (0.0)  0 (0.0) | 118 (100)  0 (0.0)  0 (0.0)  **0 (0.0)** | n. a. |

n. a.: not available; ^1^ OR of conditional (fixed-effects) logistic regression analysis with cancer (yes/no) as outcome. For each independent variable a separate model was created.
